# Improving the diagnosis of bipolar disorder through machine learning – when is the time right?^☆^

**DOI:** 10.1016/j.nsa.2025.105529

**Published:** 2025-10-01

**Authors:** Anna Luisa Klahn

**Affiliations:** Department of Psychiatry and Neurochemistry, Institute of Neuroscience and Physiology, University of Gothenburg, Gothenburg, Sweden

**Keywords:** Bipolar disorder, Major depressive disorder, Machine learning, Neuroimaging, Precision psychiatry

Psychiatric diagnostics, traditionally rooted in clinical interviews based on diagnosis criteria, benefit from decades of validation as well as a nuanced gut feeling of experienced clinicians. However, these diagnostic procedures bear the risk to fall short in complex cases. One critical challenge is the distinction between bipolar disorder and unipolar major depressive disorder. As both disorders are characterised by severe depressive episodes and the onset of bipolar disorder often presents with depressive symptoms, a correct diagnosis of bipolar disorder is mostly impossible prior to a first episode of (hypo)mania. However, an early distinction is essential as treatment strategies diverge notably between the two conditions, and misdiagnosis can result in ineffective or even harmful interventions. In this regard, emerging technologies and neurobiological markers offer a promising complementation to the diagnostic framework.

In their recent publication, Calesella et al. present a machine learning pipeline applied to multimodal neuroimaging data to differentiate between currently depressed patients with either bipolar disorder or major depressive disorder (Calesella et al., 2024). The authors report promising classification accuracies, while it is important to note that only internal validation was performed limiting the generalizability of the findings. While their prediction models based on diffusion tensor imaging reached accuracy rates between 75 and 78 %, models built on grey matter voxel-based morphometry landed at 61 % accuracy. These rates are in line with the authors’ previous, comprehensive review of machine learning studies for prediction of bipolar disorder (Colombo et al., 2022). The authors conclude that machine learning algorithms applied to multimodal neuroimaging data could offer a precision psychiatry solution to the diagnostic challenges presented by bipolar and unipolar depression.

This raises a critical and often overlooked question: when should neurobiological data be collected to support diagnostic prediction? The timing of data acquisition relative to illness stage may shape both the nature of the neural signature and its diagnostic utility. We and others have reported that brain structure is affected by (hypo)manic episodes highlighting their neurobiological scarring effect (Abé et al., 2023). In the discussed study, the authors included currently depressed patients with major depressive disorder and bipolar disorder all of whom presented with an established diagnosis. This means that patients had already experienced at least two depressive episodes or, in bipolar disorder patients, at least one hypomanic or manic episode. Furthermore, most patients were treated with mood stabilizers and/or antidepressant medication. The authors state that all prediction models were corrected for, amongst others, medication load and the number of previous mood episodes. Nevertheless, the diagnosis prediction relied on brain metrics influenced by the cumulative impact of past illness events which limits the utility in identifying bipolar disorder during its prodromal stages. Notably, different neuroimaging modalities may vary in their sensitivity to external influences and temporal dynamics. For instance, grey matter volume is known to fluctuate in response to stress, mood episodes, and medication, while white matter microstructure, as captured by diffusion metrics, may present relative temporal stability (Abé et al., 2023; Linke et al., 2020; McDonald, 2015). These distinctions are crucial when considering the timing of data collection: modalities prone to rapid change may be more informative for current symptom states, whereas more stable features might offer greater value for early or trait-level prediction. To accurately distinguish bipolar disorder from major depressive disorder as early as possible, it is essential to conduct prospective longitudinal studies that collect data both prior to illness onset and during an initial depressive episode, ideally before patients have experienced any manic or hypomanic episodes.

Such prospective longitudinal studies aiming at predicting and dissociating psychiatric diagnoses inevitably present logistical and ethical challenges, including the need for large, diverse cohorts, and long follow-up periods. While predictive studies in large, general population cohorts might be unrealistic, efforts should instead focus on high-risk populations, for instance, young individuals with a family history of bipolar disorder or undiagnosed adolescents and young adults seeking help with affective symptoms for the first time. As described in a recent review, a consensus definition of individuals at high-risk for bipolar disorder is still lacking (Keramatian et al., 2022). Some studies applied clinical criteria, such as subthreshold manic symptoms, while most studies identified young individuals at high-risk based on genetic risk and a family history of bipolar disorder. However, less than 25 % of these individuals developed any bipolar spectrum disorder. These findings provide at least two intriguing implications: firstly, we need to identify protective factors that prevent the development of bipolar disorders in high-risk individuals. Second, these findings emphasize the importance of a multimodal approach to predict the onset of bipolar disorder including neurobiological, clinical, psychosocial, and epidemiological data. Such vast efforts can only be justified by the potential benefit: a predictive tool that aids clinicians in the early identification of bipolar disorder and thereby allowing to tailor interventions and mitigate the progression of bipolar disorder and its associated morbidity.

Some studies have already started tackling this task. Uchida et al. presented a prospective study in 492 children and adolescents using machine learning based on psychometric, clinical, and cognitive data. 10 % of the children developed bipolar disorder at the 10-year follow-up which could be predicted by the algorithm with 75 % sensitivity and 76 % specificity (Uchida et al., 2022). Combining functional and structural MRI metrics with clinical measures of young individuals at risk for bipolar disorder, Bertocci et al. reported affective liability factors as an estimate for prodromal bipolar symptoms and underline the importance of multimodal data in prediction models (Bertocci et al., 2019). In a multi-site study, Mikolas et al. (2024) applied a machine learning approach on whole-brain cortical thickness metrics to classify the estimated risk for bipolar disorder in help-seeking, undiagnosed adolescents and young adults reporting accuracy rates up to 63 %. The authors underline, as well, the importance of the definition of high-risk individuals in prospective studies.

The article by Calesella et al. emphasizes the potential of machine learning applied to multimodal neuroimaging data to enhance diagnostic precision in psychiatry. While the promise of precision psychiatry is evident, the translation of these findings into everyday clinical practice still has some way to go – not least because the reported accuracy, although promising, remains below the threshold typically required for clinical decision-making. Most importantly, the timing of data collection is crucial and must be carefully attuned to the purpose of the respective prediction model. Identifying high-risk individuals through precise definitions enabled by multimodal machine learning models holds the promise of advancing early diagnosis of bipolar disorder and facilitating timely, targeted interventions.

## Funding

ALK is supported by funds of the Märta-Lundqvist's Foundation, the 10.13039/100001388Wenner-Gren Foundation, the Kurt and Ingrid Dahréns Foundation, and the 10.13039/501100003745Wilhelm and Martina Lundgren Foundation. The funders had no role in preparation, review, or approval of the manuscript or the decision to submit for publication.

## Declaration of competing interest

The authors declare that they have no known competing financial interests or personal relationships that could have appeared to influence the work reported in this paper.
